# The role that choice of model plays in predictions for epilepsy surgery

**DOI:** 10.1038/s41598-019-43871-7

**Published:** 2019-05-14

**Authors:** Leandro Junges, Marinho A. Lopes, John R. Terry, Marc Goodfellow

**Affiliations:** 1 EPSRC Centre for Predictive Modelling in Healthcare, University of Exeter, Exeter, United Kingdom; 2 Centre for Biomedical Modelling and Analysis, University of Exeter, Exeter, United Kingdom; 3 College of Engineering, Mathematics and Physical Sciences, University of Exeter, Exeter, United Kingdom; 4 Living Systems Institute, University of Exeter, Exeter, United Kingdom

**Keywords:** Computational neuroscience, Applied mathematics

## Abstract

Mathematical modelling has been widely used to predict the effects of perturbations to brain networks. An important example is epilepsy surgery, where the perturbation in question is the removal of brain tissue in order to render the patient free of seizures. Different dynamical models have been proposed to represent transitions to ictal states in this context. However, our choice of which mathematical model to use to address this question relies on making assumptions regarding the mechanism that defines the transition from background to the seizure state. Since these mechanisms are unknown, it is important to understand how predictions from alternative dynamical descriptions compare. Herein we evaluate to what extent three different dynamical models provide consistent predictions for the effect of removing nodes from networks. We show that for small, directed, connected networks the three considered models provide consistent predictions. For larger networks, predictions are shown to be less consistent. However consistency is higher in networks that have sufficiently large differences in ictogenicity between nodes. We further demonstrate that heterogeneity in ictogenicity across nodes correlates with variability in the number of connections for each node.

## Introduction

Mathematical models are increasingly being used to study the emergence of large-scale, spatiotemporal brain dynamics, which are typically recorded using techniques such as electroencephalography (EEG) or functional magnetic resonance imaging (fMRI)^1–3^. A common approach is to formulate models based upon large-scale brain networks in order to understand how dynamics are shaped by network connectivity and intrinsic node properties^4–6^. There is an extensive literature for this kind of modelling^7–11^, but a popular approach is to use ordinary or stochastic differential equations to model the temporal evolution of nodes (i.e. regions of brain tissue) in combination with an estimate of network structure and coupling equations, which define how nodes interact with one another^3,12–15^. Thus, physiological mechanisms that are incorporated into these models include the presence (or absence) and weight of large-scale connections. The choice of model for nodes can be broadly split into two categories. So-called “physiological” models incorporate intrinsic node mechanisms that are derived from the properties of large regions of brain tissue^1,9,16,17^. On the other hand, “phenomenological” models do not explicitly model the physiological mechanisms of node dynamics but represent pertinent features of brain dynamics using more abstract or canonical forms. Nevertheless, they retain mechanisms relating to network connectivity^18–22^. Both approaches have been widely used to study the emergence of large-scale brain dynamics, either to better understand healthy brain functioning or the effects of disruptions associated with neurological conditions like epilepsy^8,23–29^.

In addition to a fundamental understanding of spontaneous brain dynamics, models of the response of the brain to perturbations are also crucial in order to better understand sensory processing and responses to treatment^30,31^. For example, in order to design optimal treatments for neurological and neuropsychiatric disorders, we should seek to use models to understand the effects of treatment perturbations on healthy and abnormal brain dynamics^29^. A prototypical example is epilepsy, which affects approximately 50 million people worldwide^32^. Around a third of people with epilepsy do not respond to antiepileptic drugs or other treatments^33^, and for these people epilepsy surgery can be the only way to eliminate or mitigate seizures. However, long-term post-operative seizure freedom is only achieved in around 50% of patients^34^. In addition, many patients are not referred to surgery due to difficulties in identifying the epileptogenic focus^35^. Mathematical models of seizures have the potential to help us improve our understanding of epilepsy and provide quantitative prognoses for treatment outcome^36,37^.

In the particular case of resective surgery in epilepsy, the perturbation of interest is the removal of brain tissue, which can be approximated in models by removing nodes from networks^25^. A quantity of interest in these networks is the propensity to transition between “healthy” and “seizure” states. From a mathematical perspective, the transitions between healthy and epileptiform rhythms have been modelled using different dynamical scenarios, particularly as a bistable or as an excitable system^38,39^. Whilst in a bistable system the two attractors representing the healthy and pathological states coexist, in an excitable system the states are separated in the parameter space. However, in both systems noise and other perturbations may drive the transitions. Models based on these different dynamical mechanisms have been used to describe seizure transitions^25,40–42^, however the fundamental mechanisms underlying the emergence of ictal (seizure) oscillations in the epileptic brain are still unknown.

Recently, these models have been used in the study of the effects of resective surgery^25,42–44^. For example, Goodfellow *et al*.^25^ used a neural mass model^1^ and functional networks derived from ECoG recordings to evaluate the influence of different macroscopic cortical regions (nodes) on the overall ictogenicity of the brain network, predicting the effect of the removal of each of these nodes on the emergence of epileptiform rhythms. This work extended the concept of *Brain Network Ictogenicity* (*BNI*)^40,45^, which describes the propensity of a network to generate seizures, to account for changes in *BNI* caused by node removal. When a node is removed, the *BNI* of the remaining network can be different to that of the unperturbed network. In Goodfellow *et al.*^25^ the change in *BNI* caused by the removal of a node was termed *Node Ictogenicity* (*NI*), which quantifies how much a node influences the emergence of epileptiform activity in the brain network. This method was retrospectively validated in a cohort of epilepsy patients who underwent resective surgery, and it was shown that the framework could predict post-surgical seizure freedom with an accuracy of approximately 90%^25,46^. In addition, the peri-ictal time course of *BNI* has been shown to be able to further optimize predictions of post-operative seizure freedom^47^.

In a subsequent approach, Sinha *et al*.^43^ used a phenomenological model capable of generating transitions between a steady state and oscillations due to bistability^40,43^ to model the dynamics of network nodes. The authors showed that nodes with the shortest “escape time” between these attractors were associated with nodes that were removed during surgery. This study confirmed the findings of Goodfellow *et al.*^25^ and the two approaches were compared in Goodfellow *et al.*^46^. In another application of phenomenological models to this problem, Lopes *et al*.^42^ demonstrated that an abstraction of neural mass model dynamics, namely the Theta model^48^, could be used to approximate the neural mass formulation. Having elucidated in simulations that rich-clubs should be targeted for surgery, it was shown that functional networks derived from ECoG recordings of people with epilepsy considered for surgery contain rich-club organization, and that patients with higher proportions of rich club removed were more likely to achieve post-operative seizure control^42^.

Although the above approaches have been shown to be potentially useful, it is clear that when trying to understand the response of networks to perturbations, even if network structures are considered fixed, many different models for node dynamics may be considered. When constructing person-specific predictive models, constraining the choice of model for node dynamics is often difficult. There may even be alternative choices of parameters, or different bifurcations, within the same model that lead to plausible dynamics of interest^49,50^. One could conceive of using extended data time courses to fit the model to statistics of interest, but the necessary amount of data required for such an approach is rarely available^51^. Thus, it is crucial to better understand the ways in which network-model based predictions may vary upon different choices of node dynamics.

In this study, we examine the effect that the choice of model has on the response of a network to perturbations. We focus on the application to epilepsy surgery and therefore consider node removal perturbations and state-switching dynamics. In order to better understand the effect of choosing different dynamical models, we study a range of exemplar network structures and assess whether the response to node-removal perturbations differs depending on the choice of dynamics for the nodes. We make use of a more comprehensive analysis compared to previous studies by mapping the influence of perturbations in large windows in parameter space. We show that the existence of discrepancies between model responses depends on the specific network under consideration. In spite of the differences in complexity and fundamental mechanisms, we show that there is good agreement in ranking network nodes according to their *NI* between models when the ictogenicity is distributed heterogeneously across the network. This provides useful information for the design of decision support tools for epilepsy surgery.

## Methods

We study dynamical systems that can be described in terms of stochastic differential equations of motion for nodes and a network structure connecting the nodes. We focus on models for node dynamics that have the inherent capability of switching between states, representing the transitions to seizures in epilepsy. Rather than studying the dynamics *per se*, we focus on the effect of node-removal perturbations, in analogy with epilepsy surgery. We study a range of different network topologies, first of all by fully elucidating all networks with 3 and 4 nodes, and then by studying a sample of networks with 19 nodes, which is a typical size for networks inferred from non-invasive clinical recordings in epilepsy (scalp EEG).

### Dynamic models of epilepsy

As described in the Introduction, many different dynamic models have been proposed to simulate the sporadic occurrence of seizures in the epileptic brain^17,39^. Here we focus on three representative models that have been previously used to evaluate optimal resection strategies in epilepsy surgery^25,42,43^. In subsequent sections, we present each model.

#### Physiological model

The physiologically inspired model we use in this work is a modified version of the Jansen-Rit model^16^, which takes into account the interaction between principal neurons (pyramidal cells), excitatory interneurons, and fast and slow inhibitory interneurons. The complete set of equations is given by^25,52^:

1 $$\begin{array}{rcl}{\dot{y}}_{1}^{i} & = & {y}_{2}^{i},\\ {\dot{y}}_{2}^{i} & = & AaS\{{y}_{3}^{i}-{y}_{5}^{i}-{y}_{7}^{i}\}-2a{y}_{2}^{i}-{a}^{2}{y}_{1}^{i},\\ {\dot{y}}_{3}^{i} & = & {y}_{4}^{i},\\ {\dot{y}}_{4}^{i} & = & Aa({\xi }^{i}+{p}_{P}+{R}_{i}+{C}_{2}S\{{C}_{1}{y}_{1}^{i}\})-2a{y}_{4}^{i}-{a}^{2}{y}_{3}^{i},\\ {\dot{y}}_{5}^{i} & = & {y}_{6}^{i},\\ {\dot{y}}_{6}^{i} & = & Bb{C}_{4}S\{{C}_{3}{y}_{1}^{i}\}-2b{y}_{6}^{i}-{b}^{2}{y}_{5}^{i},\\ {\dot{y}}_{7}^{i} & = & {y}_{8}^{i},\\ {\dot{y}}_{8}^{i} & = & Gg{C}_{7}S\{{C}_{5}{y}_{1}^{i}-{y}_{9}^{i}\}-2g{y}_{8}^{i}-{g}^{2}{y}_{7}^{i},\\ {\dot{y}}_{9}^{i} & = & {y}_{10}^{i},\\ {\dot{y}}_{10}^{i} & = & Bb{C}_{6}S\{{C}_{3}{y}_{1}^{i}\}-2b{y}_{10}^{i}-{b}^{2}{y}_{9}^{i},\\ {\dot{y}}_{11}^{i} & = & {y}_{12}^{i},\\ {\dot{y}}_{12}^{i} & = & {A}_{d}{a}_{d}S\{{y}_{3}^{i}-{y}_{5}^{i}-{y}_{7}^{i}\}-2{a}_{d}{y}_{12}^{i}-{a}_{d}^{2}{y}_{11}^{i},\end{array}$$

here, *y*_1_ to *y*_11_ (odd indices) represent the excitatory and inhibitory post synaptic potential of the different cell populations, *y*_2_ to *y*_12_ (even indices) are auxiliary variables, *ξ* is Gaussian noise with zero mean and standard deviation *σ* = 1.85^25^, *S* is the sigmoid function

2 $$S(\nu )=\frac{2{e}_{0}}{1+{e}^{r({\nu }_{0}-\nu )}}$$

which converts net post-synaptic potentials into afferent firing rates, and *R*_*i*_ is the coupling term, given by

3 $${R}_{i}={\alpha }_{P}\sum _{j=1}^{N}{M}_{ij}{y}_{11}^{j}$$

where *α*_*P*_ is the global coupling strength and *M* is the adjacency matrix of the network representing the interaction between nodes (brain regions). The parameter values used to solve Eq. (1) and their biological interpretation are given in Table 1. The output of the model is given by (*y*_3_ − *y*_5_ − *y*_7_), which corresponds to the membrane potential of pyramidal cells, resulting from the interactions between three populations of interneurons, one excitatory and two inhibitory^1^.

**Table 1:** Parameter values for the Physiological model and their biological interpretation^25^.

*A*	Average excitatory gain	5 mV
*B*	Average slow inhibitory gain	44 mV
*G*	Average fast inhibitory gain	20 mV
*A*_*d*_	Gain of delayed efferent activity	3.25 mV
*a*	Inverse average time constant - excitatory feedback loop	100 s^−1^
*b*	Inverse average time constant - slow inhibitory feedback loop	50 s^−1^
*g*	Inverse average time constant - fast inhibitory feedback loop	500 s^−1^
*a*_*d*_	Inverse average time constant for delayed efferent activity	100 s^−1^
*C*_1_–*C*_7_	Connectivity constants	*C*_1_ = 135, *C*_2_ = 0.8 *C*_1_, *C*_3_ = *C*_4_ = *C*_7_ = 0.25 *C*_1_, *C*_5_ = 0.3 *C*_1_, *C*_6_ = 0.1 *C*_1_
*ν*_0_,*e*_0_,*r*	Parameters of the sigmoid function	*ν*_0_ = 6 mV, *e*_0_ = 2.5 s ^−1^, *r* = 0.56 mV^−1^

The choice of parameters in this model places the system near a saddle node on invariant circle (SNIC) bifurcation^52^, such that transitions from a fixed point (background state) to a high amplitude oscillation (epileptiform dynamics) can arise due to noise. From a dynamical viewpoint, the value of the excitability parameter *p*_*P*_ quantifies the distance from the bifurcation point and therefore contributes to the propensity of the system to transition from the background to epileptiform dynamics. This propensity is also influenced by the input from other nodes, which is quantified here by the coupling strength *α*_*P*_.

#### Theta model

As described above, the dynamic mechanism underlying transitions between states in our implementation of the Physiological model is a SNIC bifurcation. The normal form of this bifurcation is given by the Ermentrout-Kopell canonical model, also known as “Theta-Neuron” or simply “Theta” model^48,53^. Originally proposed to describe neuron firing, this model has been used to represent large-scale neural masses embedded in networks^42^. When network connectivity is incorporated, this model can be described as^42^:

4 $${\dot{\theta }}^{i}=1-{\rm{c}}{\rm{o}}{\rm{s}}({\theta }^{i})+\mathrm{[1}+{\rm{c}}{\rm{o}}{\rm{s}}({\theta }^{i})]{I}^{i}$$

where

5 $${I}^{i}={p}_{T}+{\xi }^{i}+{\alpha }_{T}\sum _{j=1}^{N}{M}_{ij}\mathrm{[1}-{\rm{c}}{\rm{o}}{\rm{s}}({\theta }^{j}-{\theta }^{s})]$$

In these equations, *θ*^*i*^ is the phase of node *i*, *ξ* is Gaussian noise with zero mean and standard deviation *σ* = 8, *M* is the adjacency matrix, *θ*^*s*^ is the steady state of a single (uncoupled) node, given by

6 $${\theta }^{s}=-\,{{\rm{c}}{\rm{o}}{\rm{s}}}^{-1}(\frac{1+{p}_{T}}{1-{p}_{T}})$$

and the parameters *α*_*T*_ and *p*_*T*_ represent the coupling strength and node excitability, respectively. For uncoupled nodes and in the absence of noise, the SNIC bifurcation takes place at *p*_*T*_ = 0. When *p*_*T*_ < 0 the stable attractor is the steady state *θ*^*s*^, while for *p*_*T*_ > 0 a limit cycle emerges and is the only stable attractor of the system. The phenomenological Theta model presents some important advantages when compared to the physiological model described in the previous Subsection. The computational cost can be significantly lower, and the reduction in dimensionality and number of independent parameters make it more straightforward to understand and control the influence of connectivity and excitability in the emergence of epileptiform dynamics^42^.

#### Bistable model

A phenomenological model that is commonly used to simulate transitions between healthy and epileptiform dynamics in brain networks is a modified version of the normal form of the subcritical Hopf bifurcation^22,40,43,54^, which we refer to here as the “bistable model”. The formulation of this model for a single node in the deterministic case is given by

7 $$\dot{z}=({p}_{B}+i\omega )z+2z|z{|}^{2}-z|z{|}^{4}$$

where *z* is a complex variable that defines the two states associated with normal and pathological activity (stable fixed point and stable limit cycle, respectively), *p*_*B*_ is the excitability parameter, and *ω* controls the oscillation frequency (we use *ω* = 20 as in Benjamin *et al.*^54^). This model has a globally stable fixed point for *p* ≤ −1 and a globally stable limit cycle for *p*_*B*_ ≥ 0. In the interval −1 ≤ *p*_*B*_ ≤ 0, the stable fixed point (*z* = 0) and the stable limit cycle ($|z{|}^{2}\mathrm{=1}+\sqrt{1+{p}_{B}}$) coexist. In this work, we represent the interaction between nodes through an additive coupling in the real part of *z*, as used in Petkov *et al.*^40^ and Sinha *et al.*^43^. For *z*^*i*^(*t*) = *x*^*i*^(*t*) + *iy*^*i*^(*t*), the model is described by

8 $$\begin{array}{c}{\dot{x}}^{i}=-\,{y}^{i}\omega +{x}^{i}({p}_{B}+\mathrm{2|}{z}^{i}{|}^{2}-|{z}^{i}{|}^{4})+\beta {\xi }_{x}^{i}+{\alpha }_{B}\sum _{j=1}^{N}{M}_{ij}{x}^{j}\\ {\dot{y}}^{i}={x}^{i}\omega +{y}^{i}({p}_{B}+\mathrm{2|}{z}^{i}{|}^{2}-|{z}^{i}{|}^{4})+\beta {\xi }_{y}^{i}\end{array}$$

*M* is the adjacency matrix, *α*_*B*_ is the coupling coefficient and *ξ* represents the Gaussian noise with zero mean and standard deviation *σ* = 1.85 (*β* = 0.01)^54^.

### Quantification of state-switching dynamics

The aim of epilepsy surgery is to remove regions of brain tissue such that the brain can no longer generate seizures. As mentioned in the Introduction, Goodfellow *et al*.^25^ introduced a model-based framework to quantify the ictogenicity of networks in terms of their propensity to generate recurrent state-switching dynamics, and thereby also quantify the reduction in seizures that would result from a model surgery. Specifically, Brain Network Ictogenicity (*BNI*) was used to quantify the propensity of a network to transition to the ictal state^40,45^. *BNI* can be evaluated in practice in different ways, but a useful method is to consider it as the average proportion of time the nodes of the network spend in the ictal state, compared to a reference period of time:

9 $$BNI=\frac{{\rm{A}}{\rm{v}}{\rm{e}}{\rm{r}}{\rm{a}}{\rm{g}}{\rm{e}}\,{\rm{t}}{\rm{i}}{\rm{m}}{\rm{e}}\,{\rm{n}}{\rm{o}}{\rm{d}}{\rm{e}}{\rm{s}}\,{\rm{s}}{\rm{p}}{\rm{e}}{\rm{n}}{\rm{d}}\,{\rm{i}}{\rm{n}}\,{\rm{t}}{\rm{h}}{\rm{e}}\,{\rm{i}}{\rm{c}}{\rm{t}}{\rm{a}}{\rm{l}}\,{\rm{s}}{\rm{t}}{\rm{a}}{\rm{t}}{\rm{e}}}{{\rm{D}}{\rm{u}}{\rm{r}}{\rm{a}}{\rm{t}}{\rm{i}}{\rm{o}}{\rm{n}}\,{\rm{o}}{\rm{f}}\,{\rm{t}}{\rm{h}}{\rm{e}}\,{\rm{r}}{\rm{e}}{\rm{f}}{\rm{e}}{\rm{r}}{\rm{e}}{\rm{n}}{\rm{c}}{\rm{e}}\,{\rm{t}}{\rm{i}}{\rm{m}}{\rm{e}}}$$

The definition above is most useful when the model of interest displays spontaneous, recurrent transitions between the healthy and ictal state, and is therefore applied in this study to the Physiological and Theta models. In the use of the Bistable model, an alternative calculation of *BNI* has been considered, which is to use the escape time from steady state to the limit cycle. Specifically, for the case of the Bistable model, we initiate the dynamics in the *z* = 0 fixed point (background state) and define the *BNI* as:

10 $$BNI=1-\frac{{\rm{Average}}\,{\rm{time}}\,{\rm{nodes}}\,{\rm{take}}\,{\rm{to}}\,{\rm{transit}}\,{\rm{to}}\,{\rm{the}}\,{\rm{ictal}}\,{\rm{state}}}{{\rm{Duration}}\,{\rm{of}}\,{\rm{the}}\,{\rm{reference}}\,{\rm{time}}}$$

**Figure 1:**
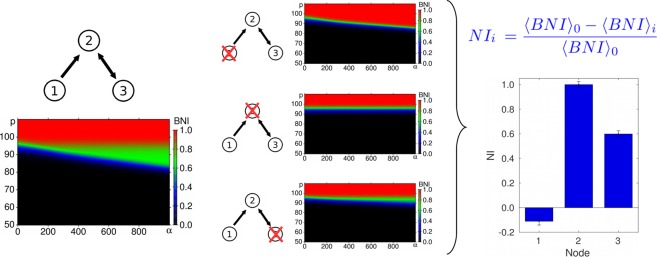
Node Ictogenicity estimation. Example of Node Ictogenicity (*NI*) calculation for a 3-node network. The diagrams show the Brain Network Ictogenicity (*BNI*) calculated for several values of the coupling (*α*) and excitability (*p*) parameters. *NI*_*i*_ represents the effect of the removal of node *i* in the network’s *BNI*.

When placed into networks, the Physiological, Theta and Bistable models display spontaneous transitions between different types of solutions for a subset of model parameters^20,25,42^. For a given network, it has been shown that these state-switching dynamics can be found by varying the global connectivity strength or the intrinsic excitability of nodes^20,25,42^. In this study, we seek to obtain a more comprehensive quantification of the presence of different states in each model. To do this, we consider the dynamics of each model over a range of values of both the connectivity strength and the intrinsic “excitability” of each node (i.e. its proximity to a bifurcation, see Fig. 1). Specifically, we define a range of values of *p* and *α*. For a given network, we simulate each model, for each parameter value over a regular grid of 192 × 192 points using an Euler-Maruyama scheme with fixed time step, optimized individually for each model (between 1 × 10^−3^ and 5 × 10^−3^) and calculate *BNI* according to Eq. (9) (or (10), for the Bistable model), for each set of parameters. The result is a two-dimensional map of *BNI* values (see Fig. 1). This approach allows a more extensive quantification of changes in ictogenicity compared to previous studies^25,42,43^, taking into account the influence of the excitability and connectivity parameters at the same time.

### Quantification of the effect of node removals

The definitions of *BNI* above provide a starting point to quantify the effect of removing nodes from networks, in a way that is pertinent for epilepsy surgery. Specifically, we define node ictogenicity (*NI*) as the change in *BNI* when a node is removed from the network:

11 $$N{I}_{i}=\frac{BN{I}_{0}-BN{I}_{i}}{BN{I}_{0}}$$

The subindices 0 and *i* refer to the complete network (before the removal of any node) and the network after the removal of node *i*, respectively. In previous work^25^, the reference state *BNI*_0_ was defined to be a state in which the unperturbed (complete) network spent half of the time in epilieptiform dynamics (*BNI*_0_ = 0.5). This value was originally chosen in order to try to optimize detection of changes in *BNI*. For a network in which the intrinsic parameters of every node are equal, such a state can be achieved by increasing the global connectivity strength from zero, until a state in which *BNI*_0_ = 0.5 is attained. Here, we use a different and more comprehensive approach, computing statistics over the 2-dimensional map of *BNI* to calculate the change in dynamics as a result of the removal of a node. Specifically, *NI* is then calculated as the percentage reduction in average *BNI* over the whole map as follows:

12 $$N{I}_{i}=\frac{{\langle BNI\rangle }_{0}-{\langle BNI\rangle }_{i}}{{\langle BNI\rangle }_{0}}$$

where the brackets 〈〉 represent the average over all simulations over the *α* × *p* diagram.

The greater the reduction in epileptiform activity after the removal of node *i*, the greater the value of *NI*_*i*_. The ictogenicity of a node can be negative if the removal of this node results in an increase in the average spiking (or a reduction in the escape time, for the Bistable model) of the remaining network.

The ranges of coupling and excitability for the three dynamical models are given in Table 2. The size of the windows in parameter space where the *BNI* is calculated for each model might influence the accuracy in capturing changes in the *BNI*, so we aimed to define regions that are large enough to include parameter values already considered in the literature, as well as to include whole regions of interest for the single node cases (like the bistability region for the Bistable model), but at the same time small enough to avoid considering wide regions where small or no changes in *BNI* are seen, consequently “diluting” the effects we wish to observe.

**Table 2:** Ranges of the excitability and coupling parameters for the calculation of the bidimensional *BNI* diagrams.

Model	Excitability	Coupling
Physiological	[50, 110]	[0, 1000]
Theta	[−4, −0.1]	[0, 10]
Bistable	[−1, 0]	[0, 10]

### Comparison of perturbation effects

For each network realization and choice of model, the distribution of *NI* was calculated according to Eq. 12 (i.e. {*NI*_*i*_}, where *i* indexes nodes in the network). In order to compare how these distributions differ for different choices of model within the same network realization, we took into account the ranking of *NI* across nodes as well as their relative values. To do this, we use a weighted Kendall rank correlation measure^42,55^, defined by:

13 $$\tau =\frac{P-Q}{P+Q},$$

where *P* (*Q*) is the number of pairs of nodes ranked in the same (inverse) order by both models. In order to quantify the differences between models in a more precise way, the sums in *P* and *Q* were weighted by the term $|N{I}_{i}^{A}-N{I}_{j}^{A}|\times |N{I}_{i}^{B}-N{I}_{j}^{B}|$, where *i* and *j* refer to the nodes being ranked, and *A* and *B* represent the different models under comparison. The weighted Kendall rank is a number in the range [−1, 1], where 1 represents total agreement and −1 represents total disagreement (rank in the inverse order) between the two compared ranks. It is important to point out that the weighted Kendall rank does not take into account nuances of the shape of the *NI* distributions being compared beyond the rank ordering of *NI* values. However, given that here we are mainly interested in identifying the most ictogenic nodes, the rank is our measure of interest.

In this work we analyze binary directed networks with three, four and nineteen nodes. We consider all 13 3-node and 199 4-node networks that are connected and nonisomorphic (the latter were obtained using the software Nauty^56^). In addition, 125 19-node random networks were selected by varying the probability of node connectivity between 0 and 1, choosing connected and nonisomorphic networks with an approximately uniform distribution of number of edges in the interval between 18 and 342 (minimum and maximum number of edges for 19-node directed connected networks).

## Results

### Networks with three and four nodes

In order to systematically analyze the role that the choice of model has on the effect of node-removals, we first studied small networks in which the full set of distinct topologies could be elucidated. There are 13 3-node networks and 199 4-node networks that are directed, connected and topologically distinct (nonisomorphic). For each of these networks, we calculated the distribution of *NI* (see Methods) for each of the three models of interest.

**Figure 2:**
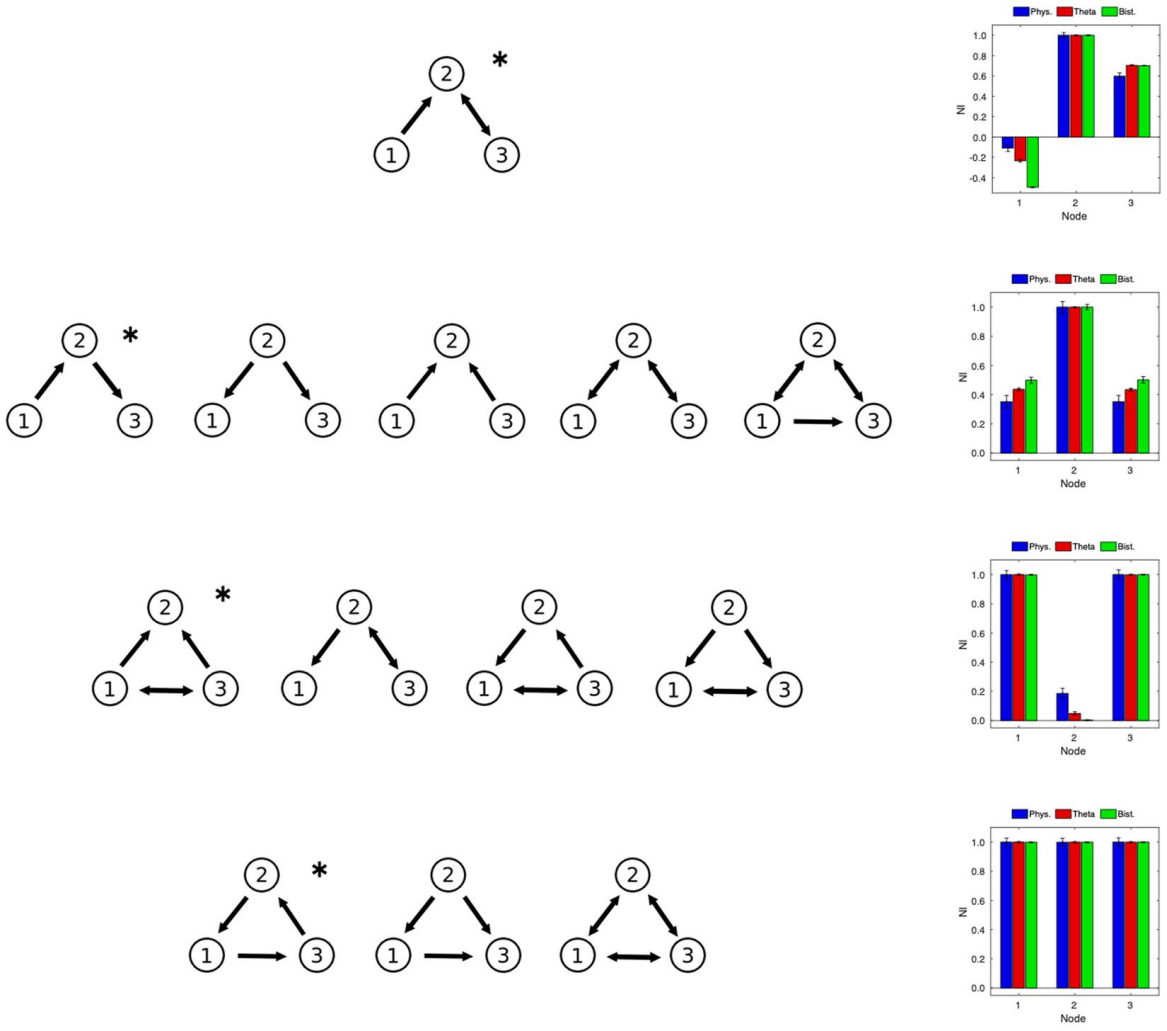
Comparison of Node Ictogenicity for 3-node networks. Normalized Node Ictogenicity (*NI*) for the thirteen 3-node nonisomorphic connected networks, calculated using three different dynamical models (see Methods). Networks are grouped by similarity in their NI distribution. An examplar distribution is presented for each group (indicated by the asterisk). See the Supplementary Information for the *NI* distribution of all networks.

As shown in Fig. 2, the resulting *NI* distributions for 3-node networks can be divided in four groups. For the first network, shown on top of Fig. 2, the three nodes have quite distinct ictogenicity, where node 1 seems to act as a “controller” of the dynamics resulting from the interaction between the other two nodes, in a way that when this node is removed, the ictogenicity of the remaining network increases. For the second group, one of the nodes (node 2) clearly has a larger *NI* than the other nodes. For the networks in this group, node 2 has a larger degree (the sum of in- and out- degrees) than the other nodes, as well as an equal number of connections to nodes 1 and 3 (Fig. 2, second row). For the third group (Fig. 2, third row), two of the nodes have relatively high *NI* (nodes 1 and 3). These networks are characterized by having only one pair of nodes that are mutually connected. Finally, for the fourth group (Fig. 2, bottom row), all nodes have a similar value of *NI*. For each network in the fourth group, all nodes have the same number of connections. In each case we observe that the distribution of *NI* is equivalent for the three models, in terms of the ranking of nodes. The similarity between model predictions for 3-node networks show that, for these networks, the models are characterizing the networks in the same way regarding the contribution of each node to the generation of epileptiform dynamics. The *NI* diagrams presented in Fig. 2 are representative examples of each of the network groups. For the individual diagrams associated with all 13 networks see Supporting Information.

**Figure 3:**
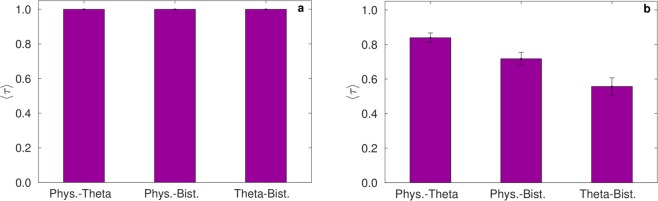
Comparison of Node Ictogenicity for 4-node and 19-node networks. Average weighted Kendall rank coefficient (〈*τ*〉) estimating the level of agreement between models. The coefficient is calculated (**a**) over all 199 4-node networks and (**b**) over all 125 19-node networks, for pairwise comparisons between the three models.

An analysis of *NI* distributions in 4-node networks is presented in Fig. 3(a). Since the number of networks in this case is large, we studied the average weighted Kendall rank (〈*τ*〉, see Methods) across all 199 topologically distinct networks. Figure 3(a) demonstrates that 〈*τ*〉 = 1 for all pairwise comparisons of the three models, indicating that, as in the case of 3-node networks, the response to node-removal perturbations is equivalent among the considered models.

### Networks with a larger number of nodes

In order to better understand the influence that the choice of model has on the effect of node removal for larger networks, which we might consider more in line with those derived from clinical data^57^, we examined the effect of this type of perturbation in a set of 19-node networks. Since the number of topologically distinct networks with 19 nodes is too large to systematically explore, given the cost of computing *NI*, we quantified *NI* for 125 sampled random networks, taking into account a broad spectrum of topologies by stratifying our sampling according to the number of edges (see Methods). The average Kendall rank 〈*τ*〉 calculated over all 125 networks is presented in Fig. 3(b). It can be seen that the Physiological and Theta models show highest concordance in ranking, having an average *τ* (〈*τ*〉) of 0.84. The Physiological and Bistable models have 〈*τ*〉 = 0.72, and the concordance between the Theta and Bistable models is lowest, with 〈*τ*〉 = 0.56. Thus in larger networks, the choice of dynamics of the individual nodes plays a more significant role than in smaller networks.

In order to investigate the reasons for this, we examined the distribution of *NI* across networks and sought to assess how this may be associated with similarities in perturbation effects across models. The results in Fig. 2 highlight that some networks give rise to heterogeneous *NI* distributions, so we asked whether the agreement between the *NI* distribution of models is related to the extent of this heterogeneity. To quantify *NI* heterogeneity we calculated, for each network and each model, the difference between the highest and lowest values of *NI* (which we denote as Δ*NI*).

**Figure 4:**
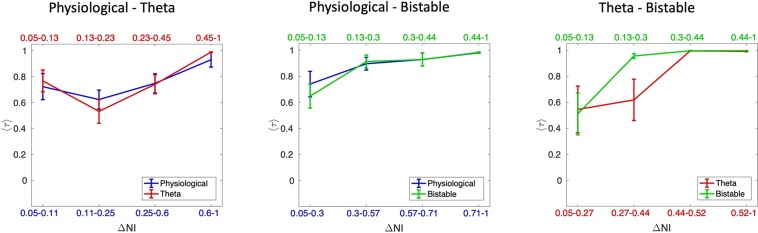
Average weighted Kendall rank as a function of the heterogeneity in the *NI* distribution. The curves show how 〈*τ*〉 changes as a function of the Δ*NI* of both models being compared. Ranges of Δ*NI* for each point are defined so that each point shows statistics calculated over the same number of networks. Error bars show the standard error of the mean.

In Fig. 4, we demonstrate how the agreement between the three models (as quantified by 〈*τ*〉) varies as a function of the heterogeneity in the *NI* distribution of each model (as quantified by Δ*NI*). In this analysis, we normalize the values of Δ*NI* across all 125 networks to values between 0 and 1, independently for each model. We then considered the networks with normalized Δ*NI* > 0.05 and split them into four equally sized bins according to their value of Δ*NI*. We used each model in the comparison pair as the reference for this, so that we could mitigate potential differences in scaling of NI for different models. For all of the three pair-wise comparisons it is clear that the more heterogeneous the NI distribution is, the better the two models being compared agree in ranking NI, independently from which model’s Δ*NI* is taken as a reference. For example, the average value of *τ* is highest, and close to a value of one, when one only considers networks in the top quartile of Δ*NI*. We further considered whether there is increased concordance in model predictions in subsets of the nodes. To this end we focused on networks with *τ* < 1 and calculated *τ* when only the 5 or 10 most ictogenic nodes are considered. Figure 4 of the Supplementary Material shows that 〈*τ*〉 is higher when only the 5 nodes with largest *NI* are considered for comparisons involving the Bistable model. However, the value of 〈*τ*〉 itself is still small in this case.

**Figure 5:**
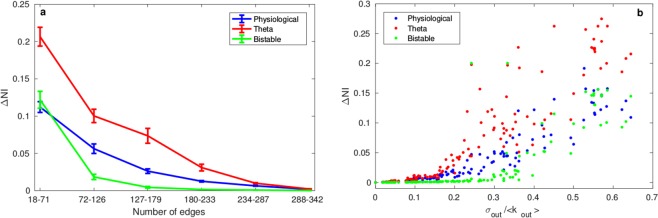
Δ*NI* as a function of global and local network measures. Δ*NI* for 19-node networks as a function of (**a**) the number of edges and (**b**) the normalized standard deviation of outdegree *σ*_*out*_/<*k*_*out*_>. Error bars show the standard error of the mean.

Since heterogeneity in *NI* is an important determinant of concordance between models, we sought to better understand whether discrepancies between *NI* distributions occurred for networks with particular topologies. Figure 5(a,b) show how Δ*NI* relates to the number of edges in each network and the normalized standard deviation of outdegree, for all four models in the case of 19 node networks. Figure 5(a) shows that the greatest differences in *NI* are observed for relatively sparse networks (low number of edges). As the networks become progressively more dense, the ictogenicity of its nodes become more homogeneous (lower Δ*NI*). Figure 5(b) indicates the presence of a nonlinear correlation between inhomogeneities in node degree (as quantified by the normalized standard deviation of outdegree) and inhomogeneities in *NI*. We quantified this correlation and found Spearman correlation coefficients between *σ*_*out*_/<*k*_*out*_> and Δ*NI* of 0.979 (Physiological), 0.961 (Theta) and 0.972 (Bistable) (*p* < 0.001 for all models). Thus, to summarize, the effect of perturbations (node removal) is dependent on the choice of model in 19-node networks, but only when *NI* is homogeneous. In contrast, networks with heterogeneous *NI*, for example those with a large difference between the minimum and maximum values of *NI* across nodes (as quantified by Δ*NI*), display good concordance in the effect of perturbations across different node dynamics. Furthermore, we find that networks with homogeneous *NI* distributions correspond to networks with low normalized degree variance and greater number of edges.

## Discussion

In this study we systematically characterized the effects that different choices of dynamical description have on predictions made using network models of epilepsy surgery. We considered three alternative models that have been used to understand the emergence of seizures in large-scale brain networks to represent the dynamics of nodes^1,10,18,25,40,42,43^. We used the measure *NI* to quantify the change in epileptiform dynamics that occurs upon removal of a node from the network. Thus, the *NI* is a prediction for the effect of epilepsy surgery. Our analysis points to the importance of *heterogeneity* of networks as a determinant of the importance of choice of model. By this we mean the extent to which properties of each node (for example the *NI*, or the degree) varies across different nodes of a network. We find that when networks are sufficiently heterogeneous, the three models we considered give equivalent rankings of nodes in terms of what happens to epileptiform dynamics when they are removed. For example, for all possible 13 3-node and 199 4-node networks, all three models produce the same ranking of nodes, in terms of their *NI* (Figs 2 and 3(a)). Therefore, for these small networks our results show that the choice of model has no influence on which nodes would be predicted to be targets for epilepsy surgery. As we consider larger networks, with 19 nodes, the choice of model dynamics can begin to cause deviations in predictions from each model, i.e. affecting the relative ranking of nodes in terms of their *NI* values. However, we find that networks with a sufficiently heterogeneous distribution of *NI*, i.e., where at least some nodes contribute very differently to the emergence of epileptiform dynamics, the level of agreement between predictions from different models improves considerably, yielding high values of the Kendall rank for all models (Fig. 4).

We further show that networks in which nodes differ significantly in *NI* (i.e. high Δ*NI*) display greater variability in node degree (Fig. 5). Since we demonstrated predictions from the three models are in better agreement when the *NI* distribution is heterogeneous, this suggests that networks that contain some highly connected nodes (compared to the average), will present an *NI* distribution which is robust to the choice of model used to calculate it. This result corroborates and extends the findings of Lopes *et al*.^42^, where the authors show that the Physiological and Theta models present good agreement in ranking nodes according to their *NI* values for random networks of 15, 30 and 50 nodes, and higher agreement in 50 nodes scale-free networks (which are highly heterogeneous networks).

The contribution of each individual node to the emergence of epileptiform dynamics is, in general, dependent on the size of the network. The larger the network is, the smaller contribution we expect each node to make to the emergence of epileptiform dynamics, which can lead to a more homogeneous distribution of *NI*. If differences in *NI* between nodes are very small their rank ordering could be more susceptible to noise, and therefore less robust. This is a contributing factor to why we observe reduced concordance between predictions of models when *NI* is homogeneous (low Δ*NI*). From a practical perspective, we are predominantly interested in identifying which brain regions (nodes) are the most ictogenic for the purpose of recommending these for surgical resection. Such regions would be more readily distinguishable if the *NI* distribution is sufficiently heterogeneous. Our results suggest that predictions from the three considered models are concordant when there are nodes that are clearly more ictogenic than others within the network. Recent studies demonstrate that in fact both functional^42^ and structural^58^ brain networks derived from epilepsy patients display such network heterogeneity (as demonstrated, for example, by the presence of hub nodes), thus suggesting that predictions from the three models should be concordant when applied to real brain networks.

It is important to point out that the distribution of *NI* for any given model emerges from an interplay between the node dynamics and the network structure. One can envisage extremes in which the network structure plays a very large or very small role in the emergent dynamics. Networks with homogeneous connectivity (i.e. low standard deviation in degree, as shown in Fig. 5) present a case in which the connectivity of the network does little to facilitate differences in the dynamics of different nodes. Therefore, the distribution of *NI* is homogeneous (as shown in Fig. 5) and the largest determinant of the dynamics of the network is the choice of model for the nodes. On the other hand, networks with heterogeneous connectivity are examples for which the network structure itself places constraints on the emergent dynamics of nodes since, for example, there will be nodes that can heavily influence the dynamics of other nodes due to the nature of their connections. In this case, the network topology, rather than the choice of model, plays the biggest role in determining the dynamics of the network.

We also show that Δ*NI* decreases as the number of edges in networks increases. This would suggest that a greater level of agreement between predictions of models is obtained for sparse networks. Networks with fewer edges have heterogeneous, rather than homogeneous, connectivity (the extreme case of homogeneous connectivity has full connectivity, i.e. the maximum number of edges). In clinical applications that have used functional connectivity networks, thresholding or surrogate methods are typically used to focus on the most significant functional connections^25,59,60^, which can yield sparse networks. This is mainly justified to avoid spurious connections due to indirect correlations or random effects. Hence, we might expect large-scale brain networks derived from clinical data to yield predictions that are robust to the choice of model.

In terms of the methodology we employed, an advance in the current work is the more comprehensive approach we used to calculate *BNI* and *NI*, where changes in dynamics due to both changing node excitability and network connectivity are taken into account. Such an approach avoids setting specific arbitrary choices for parameters. Nevertheless, this method depends on the choice of boundaries for these parameters. It is impossible to define *a priori* an optimal parameter window in which heterogeneity in the *NI* distribution is maximized for all models, and a systematic search for such a window would be an extremely demanding numerical task. Our strategy was to balance the definition of boundaries in order to include all the dynamical changes of interest (i.e. both “healthy” and epileptiform dynamics). In the future, machine learning algorithms and other advanced statistical tools could be used to search for windows that optimize *NI* heterogeneity^50^. In addition, it is interesting to note that, in spite of the Bistable model being analyzed using a different definition of *BNI* (see Methods), the *NI* distributions closely matched those obtained with the Physiological and Theta models. This shows that the obtained results are not only robust across different models, but also across alternative definitions of ictogenicity.

Despite various attempts to derive mathematical models for the generation of seizures^1,18,29,38^ there is still a lack of understanding regarding which dynamical description should be used to represent node dynamics in models of ictogenic networks. Indeed, it is likely that different dynamic descriptions are appropriate for different patients. In order to develop a deep understanding about the fundamental mechanisms generating the dynamics observed in real brain networks, and also to optimize these methods for individuals, the understanding of how alternative descriptions compare is paramount. The present work addresses this by clarifying under what circumstances alternative dynamical models yield different predictions for the effect of removing nodes from a network, as quantified by *NI*.

Comparisons between different models of node dynamics have been performed in other contexts. Messe *et al*.^61^ compared seven different computational models, showing that the simplest model considered (simultaneous autoregressive model) performed better than the other more sophisticated dynamical descriptions in predicting functional connectivity from structural connectivity using DWI and MRI. In the wider context of systems biology, many efforts have been recently undertaken to simplify complex models of biochemical reaction networks^62,63^. Santolini and Barabasi^64^ analyzed 87 biological models and showed that “Dynamics-Agnostic Network Models”, a framework based exclusively on network topology, can provide 65–80% accuracy in predicting the impact of specific perturbation patterns when compared to the complete biochemical model. This result highlights the importance of understanding the relationship between network topology and model dynamics. In our study we focussed on node-removal perturbations and demonstrated that whether choice of model is important depends on network topology. In practice, if a network under consideration is sufficiently heterogeneous, our analysis shows that we could use any of the three models studied, and the criterion used to choose between the three could be computational efficiency. Thus the lower dimensional theta or bistable models would be preferable.

Furthermore, a comprehensive understanding of the effects of alternative dynamical descriptions on the spontaneous activity of large-scale networks can help provide additional insights into other more general modelling frameworks used to study responses to perturbations. Computational modelling techniques have been used to optimize targeting in Deep Brain Stimulation (DBS)^65^, to explain changes in brain rhythms induced by Transcranial Magnetic Stimulation (TMS)^66^, and to estimate the influence of electrode displacement on Transcranial Direct Current Stimulation (tDCS)^67^, to mention a few examples. These perturbation techniques are becoming increasingly popular in the treatment of several neurological and neuropsychological disorders, including epilepsy^68–70^. We hope the present work can serve to highlight the importance of understanding the influence of using alternative dynamical descriptions in predictive modelling, and can serve as a basis for this type of systematic analysis.

## Supplementary information

Supplementary Information
