# Supplementary material for: The role that choice of model plays in predictions for epilepsy surgery
Source: Sci Rep. 2019 May 14;9:7351. doi: 10.1038/s41598-019-43871-7 (PMC6517411; doi:10.1038/s41598-019-43871-7)
Supplement: Supplementary file 1 — Supplementary Information [file 41598_2019_43871_MOESM1_ESM.pdf]

The role that choice of model plays in predictions for epilepsy surgery

Leandro Junges[1,2,3,4,*], **Marinho A. Lopes**[1,2,3,4], **John R. Terry**[1,2,3,4,+], **and Marc Goodfellow**[1,2,3,4,+]

[1]EPSRC Centre for Predictive Modelling in Healthcare, University of Exeter, Exeter, United Kingdom
[2]Centre for Biomedical Modelling and Analysis, University of Exeter, Exeter, United Kingdom
[3]College of Engineering, Mathematics and Physical Sciences, University of Exeter, Exeter, United Kingdom
[4]Living Systems Institute, University of Exeter, Exeter, United Kingdom
[*]l.l.l.junges@exeter.ac.uk
[+]These authors are joint senior authors on this work

Supplementary Figures

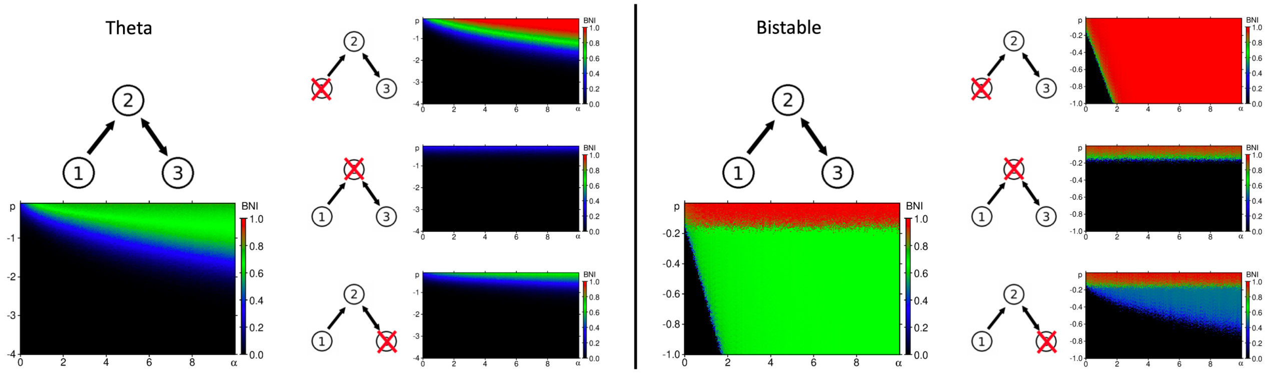

Figure 1. Calculation of node ictogenicity for a 3-node network using the Theta model and the Bistable model.
The diagrams show the Brain Network Ictogenicity (*BNI*) calculated for several values of the coupling (α) and excitability (p) parameters. NI_i represents the effect of the removal of node i in the network's *BNI*. The respective *NI* distributions can be found in Fig. 2D of the Supplementary Information.

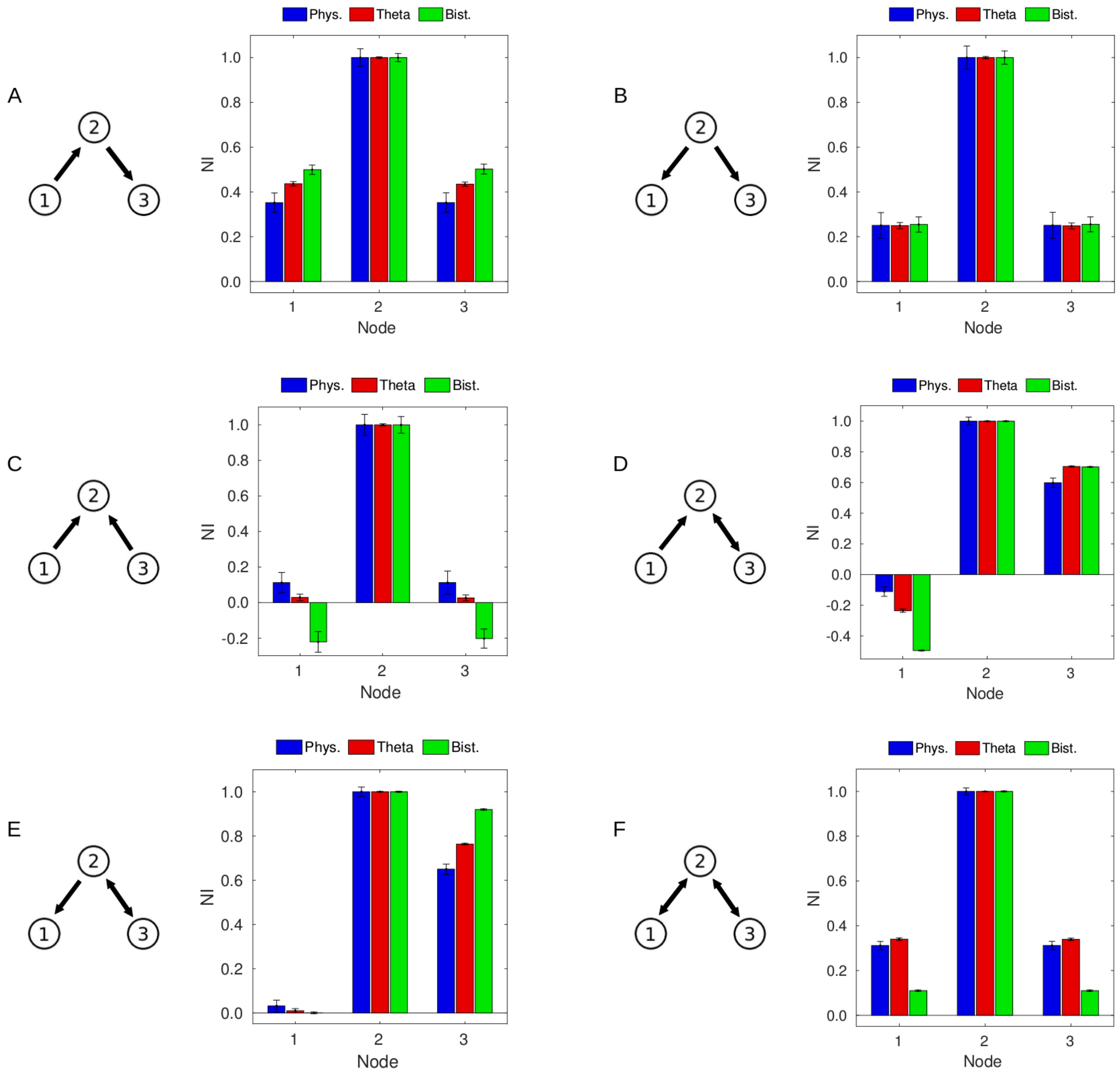

Figure 2. Comparison of Node Ictogenicity for all 13 3-node nonisomorphic connected networks. Normalized *NI* calculated using three different dynamical models. Note that the ranking of nodes according to their *NI* value is the same for every network, regardless of the model (remaining networks in Fig 3).

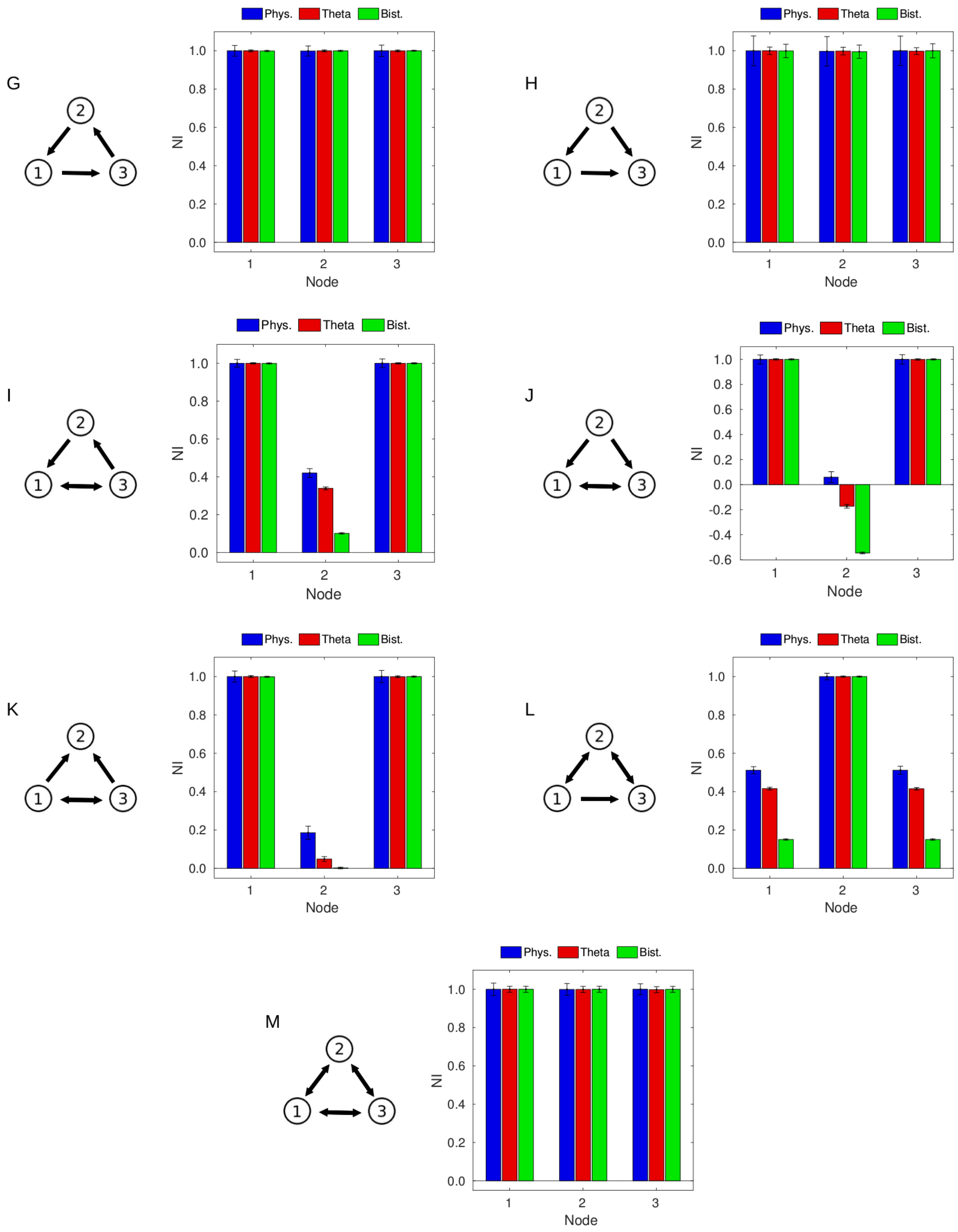

Figure 3. **[Cont.] Comparison of Node Ictogenicity for all 13 3-node nonisomorphic connected networks.**
Normalized *NI* calculated using three different dynamical models. Note that the ranking of nodes according to their *NI* value is the same for every network, regardless of the model.

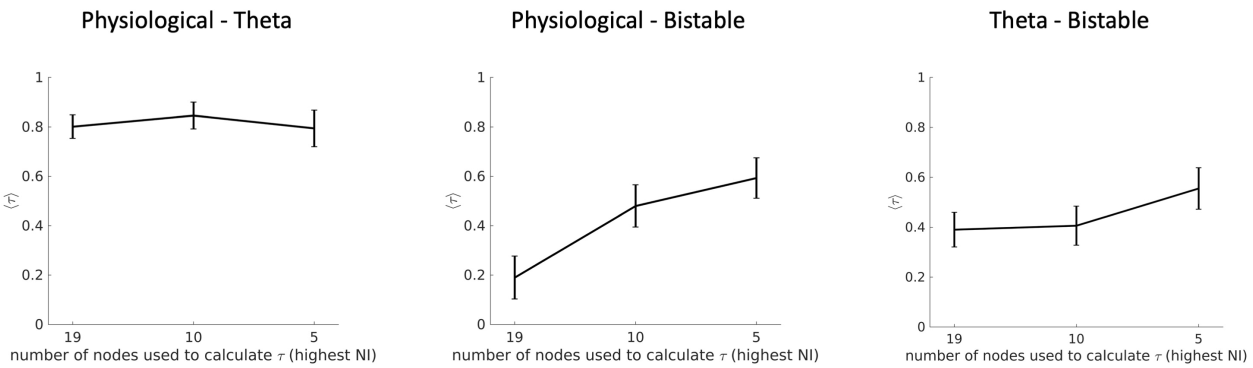

Figure 4. Average Kendall rank for different number of nodes (with highest NI).
Calculation of the average Kendall rank ($\langle\tau\rangle$), for the cases where $\tau < 1$, considering the 5, 10 and 19 most ictogenic nodes, using one of the models being compared as a reference.

Supplementary Data

In order to guarantee reproducibility of the results presented in this paper, the authors make available a MATLAB file with all the networks with four and nineteen nodes used here (the networks with three nodes are described in the figures above). This information, in addition to the parameter values and numerical details provided in the paper, should be sufficient for reproducibility.
